# Functional and metabolic dichotomy of murine γδ T cell subsets in cancer immunity

**DOI:** 10.1002/eji.201948402

**Published:** 2020-12-08

**Authors:** Noëlla Lopes, Bruno Silva‐Santos

**Affiliations:** ^1^ Instituto de Medicina Molecular João Lobo Antunes, Faculdade de Medicina Universidade de Lisboa Lisbon Portugal

**Keywords:** γδ T cells, tumor immunology, IFN‐γ, IL‐17, metabolism

## Abstract

γδ T cells can display a plethora of immune functions, but recent studies have highlighted their importance, in multiple disease models, as sources of the pro‐inflammatory cytokines, IL‐17A (IL‐17), and IFN‐γ. These are produced by distinct murine effector γδ T cell subsets that diverge during thymic γδ T cell development. Among the multiple roles these subsets play in peripheral tissues, a striking dichotomy has emerged at tumor sites: whereas IFN‐γ^+^ γδ T cells inhibit tumor cell growth, IL‐17^+^ γδ T cells promote tumor progression and metastasis formation. In this review, we discuss the main lines of evidence, mostly from preclinical studies in mouse models, for this functional dichotomy in cancer immunity. We further highlight very recent advances in our understanding how metabolic sources and pathways can impact on the balance between IFN‐γ^+^ and IL‐17^+^ γδ T cells in the tumor microenvironment, which opens a new exciting avenue to explore toward the application of γδ T cells in cancer immunotherapy.

## Graphical abstract

γδ T cell subsets have opposite roles in cancer immunity: whereas IFN‐γ^+^ γδ T cells inhibit tumor cell growth, IL‐17^+^ γδ T cells promote tumor progression and metastasis formation. Furthermore, metabolic sources and pathways can impact on the γδ T cell subset balance in the tumor microenvironment. Manipulating γδ metabolism may pave the way toward the development of novel immunotherapies.

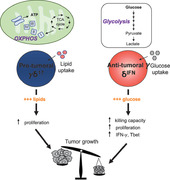

## Introduction

γδ T cells are unconventional T cells that express a unique T‐cell receptor (TCR) composed of γ and δ chains. Although they are of low abundance in lymphoid organs, γδ T cells can play key roles in the physiology and immune surveillance of many tissues, as firmly demonstrated in mouse models [1]. In mice, γδ T cell development in the thymus involves a specific sequence of somatic rearrangement of TCR genes during ontogeny, leading to subset‐characteristic TCRγ variable region (Vγ) usage [2]. Importantly, these thymic subsets are intrinsically biased to produce either IL‐17A (IL‐17) or IFN‐γ as result of a complex process of “developmental pre‐programming” that involves TCR‐dependent and TCR‐independent mechanisms, as we [3] and others [2, 4] have reviewed elsewhere.

Distinct effector γδ T cell subsets can be defined based on the expression of the TNF‐receptor superfamily member, CD27 [5], and further resolved using additional markers such as CD44 and CD45RB [3, 6, 7]. The fate of γδ T cell progenitors is seemingly dictated by TCRγδ signaling, since strong TCRγδ signals inhibit the generation of IL‐17‐producing γδ (γδ^17^) T cells while promoting IFN‐γ‐producing γδ (γδ^IFN^) T cell development [3, 6, 7]. The cellular and molecular mechanisms underpinning the differentiation of γδ^17^ versus γδ^IFN^ T cells have been recently reviewed elsewhere [8].

Effector γδ T cell subsets are known to play crucial roles in tissue homeostasis [1], autoimmunity [9], and cancer [10]. Here, we will concentrate on cancer immunity, where γδ T cells play crucial roles and thus represent a promising option for next‐generation immunotherapies [10, 11]. In this context, γδ T cells share functional similarities with their αβ T cell counterparts, such as cytotoxic functions and secretion of pro‐inflammatory cytokines. However, in contrast to αβ T cells, γδ T cells are mostly independent on MHC‐mediated presentation of mutated antigens, although a recent study described an exception where specific melanoma‐associated antigens were recognized by human γδ T cells in an MHC class I‐restricted manner [12]. In general, γδ T cells sense “stress‐inducible” changes, conveyed either via the TCR‐γδ or NK cell receptors, like NKG2D, to become activated and deploy rapid effector responses in transformed tissues [13, 14].

In the tumor microenvironment (TME), murine γδ T cells typically belong to the Vγ1^+^, Vγ4^+^, or Vγ6^+^ (using the Heilig & Tonegawa nomenclature [15]) subsets. These subpopulations have different functional biases to produce either IL‐17 or IFN‐γ: Vγ1^+^ T cells are IFN‐γ‐biased, whereas Vγ6^+^ T cells produce almost exclusively IL‐17, and Vγ4^+^ T cells contain sizeable IL‐17‐producing and IFN‐γ‐producing sub‐populations [2, 3]. This is particularly important because these two cytokines, and thus the corresponding γδ T cell subsets, typically play opposing roles in the TME: whereas γδ^IFN^ cells are potent antitumor type 1 cytotoxic effectors, γδ^17^ cells are mostly pro‐tumoral, through inducing angiogenesis, tumor cell proliferation, and recruiting immunosuppressive cells to the TME [10]. In this review, we discuss the current knowledge on tumor‐associated γδ T cell subsets, while integrating very recent discoveries on a striking metabolic dichotomy between γδ^IFN^ and γδ^17^ T cells [16] that define a new perspective to improve the performance of γδ T cells in cancer immunotherapy.

## Pro‐tumoral functions of γδ^17^ cells

**Figure 1:**
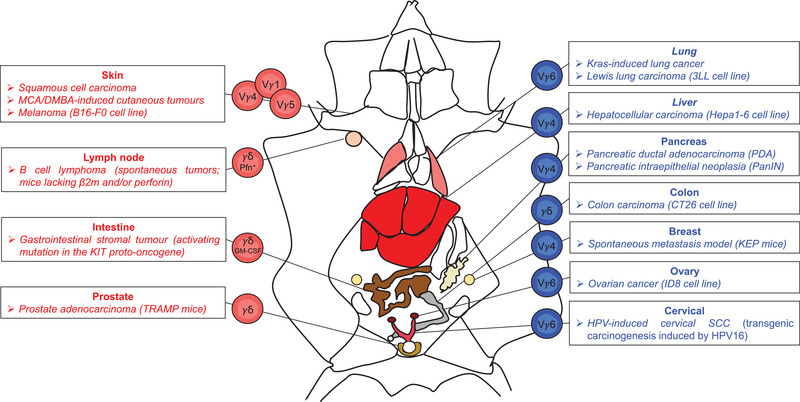
Opposite roles of murine γδ T cell subsets in cancer. Distinct γδ T cell subsets play an anti‐ (*red*) or pro‐ (*blue*) tumoural roles in various experimental mouse cancer models. Vγ1^+^, Vγ4^+^ and Vγ5^+^ T cell subsets producing IFN‐γ are protective in several skin cancers such as squamous cell carcinoma, 3‐methylcholanthrene (MCA)/ 7,12‐dimethylbenz]alpha]anthracene (DMBA)‐induced cutaneous tumors or B16‐F0 melanoma. γδ T cells are also overtly anti‐tumoural in: (i) spontaneous B cell lymphoma in mice lacking beta 2 microglobulin (β2m) and/ or perforin (Pfn), (ii) gastrointestinal stromal tumors due to activating mutation in the KIT proto‐oncogene, via GM‐CSF, and (iii) prostate adenocarcinoma in TRAMP mice. In contrast, Vγ6^+^ γδ^17^ T cells play pro‐tumor roles in Kras‐induced lung cancer and Lewis lung carcinoma, as well as in ID8 ovarian cancer and in Human papillomavirus (HPV)‐induced cervical squamous cell carcinoma (SCC) models. Other IL‐17‐producing γδ T cells, especially Vγ4^+^ T cells, are detrimental in (i) Hepa1‐6 hepatocellular carcinoma, (ii) pancreatic adenocarcinoma in PDA and pancreatic intraepithelial neoplasia (PanIN) models, and (iii) KEP breast cancer metastatic mouse model.

**Figure 2:**
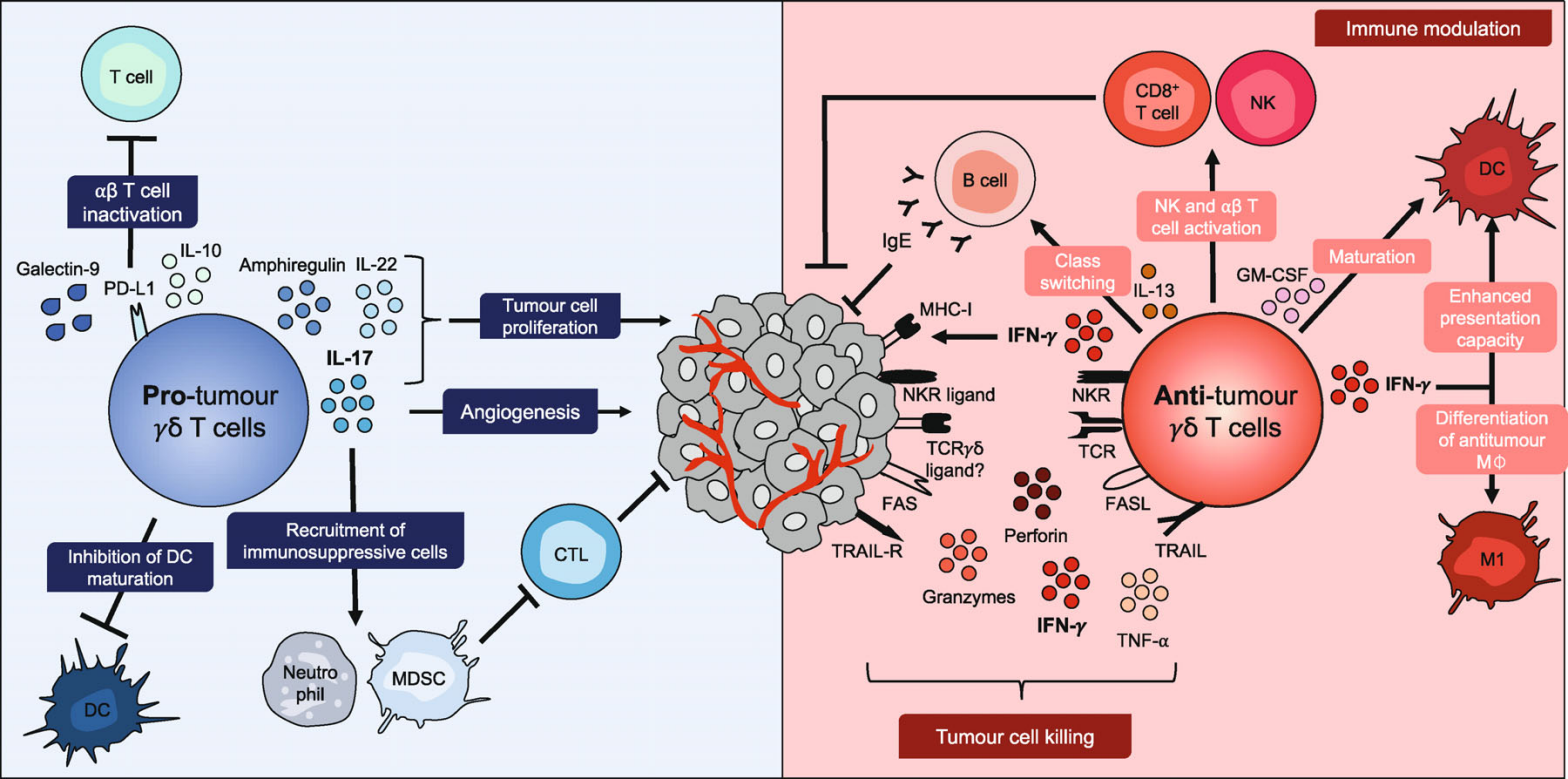
Pleiotropic functions of γδ T cells in the TME. Pro‐tumoural roles of γδ T cells (*left panel in blue*) include the production of IL‐17 which stimulates tumor cell proliferation, induces angiogenesis, and recruits immunosuppressive cells like myeloid‐derived suppressive cells (MDSC) that inhibit cytotoxic T lymphocytes (CTL). γδ T cells can also inhibit DC maturation and exert IL‐17‐independent pro‐tumor roles, via galectin‐9, programmed cell death protein 1 ligand 1 (PDL‐1) or IL‐10, which inhibit ⍺β T cell responses; or through production of amphiregulin or IL‐22 that promote tumour cell proliferation. Antitumoral roles of γδ T cells (right panel in red), activated through the T cell receptor (TCR) and NK cell receptors (NKRs) to kill tumor cells upon expression of perforin and granzymes, FAS ligand (FASL) and tumor necrosis factor‐related apoptosis‐inducing ligand (TRAIL), as well as the cytokines tumor necrosis factor ⍺ (TNF‐⍺) and IFN‐γ. IFN‐γ upregulates MHC class I expression on tumor cells, which enhances their recognition by cytotoxic T lymphocytes. IFN‐γ also induces the differentiation of antitumor macrophages (M) and enhances the presentation capacity of antigen‐presenting cells. Additionally, γδ T cells promote the maturation of DCs via secretion of GM‐CSF; promote activation of CD8^+^ T cells; increase cytotoxicity of NK cells via CD137L expression; and, through IL‐13 secretion, induce antibody class switching in B cells to autoreactive antitumor IgE.

The TME is often conducive to IL‐17 production. In fact, high levels of IL‐17 have been detected in the different types of tumor in mice and humans [17, 18]. Interestingly, it has been recently found that increased IL‐17 in the LN microenvironment during ageing leads to accumulation of γδ^17^ T cells, which associates with accelerated tumor growth [19]. Two γδ^17^ T cell subsets can be major sources of IL‐17 at tumor sites: Vγ4^+^ γδ^17^ T cells predominate in hepatocellular carcinoma [20] and in breast tumor murine models [21], whereas Vγ6^+^ γδ^17^ T cells are enriched in lung [22], ovarian [23], and cervical [24] tumor mouse models (Fig. 1). Various cytokines have been associated with γδ^17^ T cell accumulation in different tumor models: IL‐1β in breast and lung metastasis [21, 25], IL‐6 in pancreatic cancer [26], IL‐1β plus IL‐23 in hepatocellular carcinoma [20], IL‐6/IL‐23/TGF‐β in fibrosarcoma and skin carcinoma [27], and IL‐1β / IL‐6 /IL‐7 in ovarian cancer [23] models. These γδ^17^ T cell expansions and IL‐17 secretion can have three major consequences that benefit tumor progression: promotion of angiogenesis, stimulation of tumor cell proliferation, and orchestration of an immunosuppressive TME (Fig. 2). Of note, while γδ^17^ T cells are rare in humans, some studies revealed that γδ T cells can constitute a major source of IL‐17 within tumor‐infiltrating lymphocytes, particularly in colorectal cancer [28, 29].

### Angiogenesis and tumor cell proliferation

Wakita and colleagues proposed for the first time, in a seminal paper, that γδ^17^ T cells supported tumor growth through induction of angiogenesis [27]. They showed that IL‐17 within tumors was predominantly produced by γδ T cells, and in the transplantable methylcholanthrene (MCA)‐induced CMS‐G4 tumor cell line model, mice lacking IL‐17 showed reduced tumor growth associated with decreased vascular density within the tumor tissue. Conversely, the administration of IL‐17 increased the expression of pro‐angiogenic factors such as vascular endothelial growth factor (VEGF) and angiopoietin 2 by tumor cells [27]. Our group found that γδ^17^ T cells can also induce angiogenesis indirectly by upregulating proangiogenic factors in small peritoneal macrophages (SPMs) that are mobilized by IL‐17 to the peritoneal cavity in the transplantable ID8 ovarian cancer model [23]. Moreover, a more recent study showed that γδ^17^ T cells stimulated the formation of blood vessels in the dermis underlying human papillomavirus‐induced tumor lesions [24]. Interestingly, γδ^17^ T cells can also promote the formation of lung metastases through tumor‐endothelial transmigration by acting on blood vessel permeability and upregulating the expression of adhesion molecules such as E‐selectin and VCAM‐1 by endothelial cells [30].

Importantly, γδ^17^ T cells, via IL‐17, can also directly stimulate tumor cell growth. IL‐17 (provided by γδ^17^ and CD4^+^ Th17 cells) is required for the initiation and progression of early pancreatic cancer: pancreatic cells sense IL‐17 in the TME through IL‐17 receptors and downstream signaling leads to the acceleration of pancreatic intraepithelial neoplasia [26].

### Recruitment of immunosuppressive cells to the TME

γδ^17^ T cells can drive cancer progression by recruiting pro‐inflammatory or immunosuppressive cells to the tumor site (Fig. 2). He et al. showed that IL‐17 is necessary for both the recruitment and development of myeloid‐derived suppressor cells (MDSCs) [31]. We also found that γδ^17^ Vγ6^+^ T cells mobilize pro‐inflammatory SPMs expressing high levels of IL‐17RA, which in turn support ID8 ovarian cancer cell growth [23]. γδ^17^ Vγ4^+^ T cells also exert their pro‐tumoural functions by recruiting MDSCs in hepatocellular carcinoma, but in this case indirectly via induction of CXCL5 production by tumor cells [20]. This underpins an interesting positive feedback loop, since IL‐17‐stimulated MDSCs secrete IL‐1β and IL‐23 that support γδ^17^ Vγ4^+^ T cell expansion. Of note, human γδ^17^ T cells have also been associated with intra‐tumor infiltration and accumulation of MDSCs in colorectal cancer patients [28].

γδ^17^ T cells also recruit neutrophils to the TME (Fig. 2), and neutrophil abundance in metastatic breast cancer patients correlates with decreased survival [32]. In pre‐clinical spontaneous breast cancer murine models, Coffelt and colleagues demonstrated that γδ^17^ T cells drive the systemic expansion and polarization of neutrophils towards a CD8^+^ T cell‐suppressive phenotype, and thereby promote lung and LN metastasis [21, 33]. Consistently, the absence of γδ T cells or neutrophils substantially reduced pulmonary and LN metastases. However, neutrophils can display pleiotropic and somewhat contradictory roles in different cancer models. For example, our group found that tumor‐associated neutrophils can suppress γδ^17^ T cell responses through induction of oxidative stress (via ROS production), to which γδ^17^ T cells are particularly susceptible due to very low levels of the cellular antioxidant, glutathione [34]. Thus, the functional cross‐talks between γδ^17^ T cells and other immune cells, especially of the multifaceted myeloid lineage, deserves further investigation.

### Antitumor functions of γδ^(IFN)^ T cells

Girardi *et al*. demonstrated, for the first time, that γδ T cells were host‐protective in transplantable squamous cell carcinoma and methylcholanthrene (MCA)‐induced or dimethylbenzanthracene (DMBA)‐induced cutaneous tumor models, since tumor growth and progression were markedly increased in *TCRδ* ^–/–^ mice [35, 36]. Since then, γδ T cells have been shown to provide tumor immunosurveillance in various other models [10, 37], also when adoptively transferred into mice bearing B16‐F0 melanomas [38] or adenocarcinomas of the prostate (TRAMP mice) [39] (Fig. 1). By generating BM chimeras and foetal liver reconstitutions varying only in their γδ^IFN^ T cell composition, Gao *et al*. showed that mice with IFN‐γ‐deficient γδ T cells have higher tumor incidence than those bearing γδ^IFN^ T cells [40]. Following this original study, many others followed in attributing crucial antitumor roles to γδ^IFN^ T cells [10, 37, 38], which are endowed with potent cytotoxic functions and orchestrate an overt antitumor TME (Fig. 2).

### Cytotoxicity against tumor cells

The seminal studies by Girardi *et al*. focused on Vγ5^+^ dendritic epidermal γδ T cells (DETCs), which are prototypic γδ^IFN^ T cells due to thymic “developmental pre‐programming” [3, 41]. DETCs were shown to deploy potent cytotoxicity against squamous cell carcinoma, upon engagement of their signature TCR as well as the critical NK cell receptor, NKG2D [35, 36, 42]. Interestingly, recent studies have shown that rapamycin, which suppresses mTOR signaling, increases NKG2D expression on Vγ4^+^ γδ T cells, enhancing their cytotoxicity against multiple tumor cell lines [43]. Cytotoxicity is a common feature of all murine γδ^IFN^ T cell subsets, but some finer differences seem to exist. For example, *TCRδ* ^–/‐^ mice reconstituted with Vγ4^+^ γδ T cells were better protected against B16 melanoma and YAC‐1 cell lymphoma cells than those reconstituted with Vγ1^+^ γδ T cells [38].

Human γδ T cells also combine IFN‐γ production with cytotoxicity, and have been shown to kill renal cell carcinoma [44], squamous cell carcinoma [45], colon cell carcinoma [45, 46], and acute [47] and chronic myeloid leukemia cells [48], among various other tumor types [49].

The cytotoxic mechanisms are multi‐layered, involving the perforin–granzyme axis, but also “death ligands,” namely FASL and TRAIL, which engage the “death receptors” FAS (CD95) and TRAIL [39, 48, 50, 51] (Fig. 2). Of note, human γδ T cells also express CD16 (or Fcγ receptor III) that binds the Fc region of IgG antibodies, thus enabling antibody‐dependent cellular cytotoxicity (ADCC) coupled to Granzyme production and IFN‐γ secretion [52, 53, 54].

### Modulation of antitumor immunity

An important role of γδ^IFN^ T cells is to promote an antitumor TME, for which the impact of IFN‐γ on MHC class I expression makes a key contribution. For example, tumor‐infiltrating γδ^IFN^ T cells were shown to regulate (via IFN‐γ production) the expression of MHC class I on murine B16 melanoma cells, thus promoting their recognition by CD8^+^ T lymphocytes [55] (Fig. 2).

However, not all protective effects of γδ T cells in cancer immunity depend on IFN‐γ production. For example, Strid and colleagues demonstrated that DETCs promote antibody class switching to IgE, which is highly protective against chemically induced skin carcinogenesis mouse model [56]. Moreover, DETCs cross‐communicate with epithelial cells via the production of IL‐13 that activates a very dynamic epithelial stress response in the epidermis in mice, which is protective against cutaneous carcinogenesis [57]. This host‐defensive type 2 (atopic) response by murine DETCs is deployed upon sensing of NKG2D ligands during cutaneous epithelial stress [58]. It is still unclear if other γδ T cell subsets can similarly engage an IL‐13/ IgE pathway in antitumor immunity.

A recent study described a protective role for γδ T cells in a mouse model of gastrointestinal stromal tumor through the secretion of GM‐CSF [59]. The authors proposed a model where tumor‐associated macrophages secreting IL‐1β promoted GM‐CSF production by γδ T cells, leading to the maturation of CD103^+^CD11b^–^ DCs, which were associated with effector CD8^+^ T cell infiltration within tumors. In humans, the main circulating γδ T cell subset, Vγ9Vδ2 T cells, have been shown to promote CD8^+^ T cell activation based on their DC‐like ability to cross‐present antigens on MHC class I and to express high levels of costimulatory molecules upon activation [60, 61, 62, 63, 64].

In sum, γδ T cells can display multifaceted antitumor mechanisms, with type 1 cytotoxic γδ^IFN^ T cells taking center stage (Figs. 1 and 2). The functional dichotomy between (protective) γδ^IFN^ and (pathogenic) γδ^17^ T cell subsets highlights the importance of their balance in tumor immunity. In this context, we have very recently unraveled how metabolic sources and pathways differentially impact on γδ^IFN^ *versus* γδ^17^ T cells and their activities in the TME [16].

### Metabolic regulation of γδ T cell subsets in cancer

Until recently, the information on γδ T cell metabolism was very scarce. By contrast, many studies on αβ T cells had shown that metabolic pathways and metabolites regulate T cell signaling, survival, differentiation, and function [65]. Metabolism is highly dynamic in the life cycle of T cells: naïve T cells display a metabolic quiescent phenotype and use the available nutrients to maximize energy production through oxidative phosphorylation (OXPHOS) in mitochondria [66]; upon activation, T cells require a metabolic reprogramming in which they engage aerobic glycolysis, i.e conversion of glucose into lactate, in the cytoplasm [67]. While less efficient than OXPHOS to produce ATP, aerobic glycolysis generates crucial metabolic components, like glucose and lactate, important for cell growth and proliferation [68]. Further changes in metabolism also occur during the differentiation of memory T cells [69, 70], which primarily use OXPHOS, but undergo a glycolytic switch when deploying their rapid effector functions [71, 72, 73]. Our recent data demonstrate that γδ^IFN^ and γδ^17^ T cells also differentially engage in aerobic glycolysis versus mitochondrial respiration [16].

### Metabolic dichotomy in γδ T cell subsets

We investigated the metabolic profile of γδ T cell subsets using a newly developed protocol, SCENITH^TM^ (*Single Cell mEtabolism by profilIng Translation inHibition)*, which is a simple method for deciphering the complex energy systems employed by immune cells [74]. This method uses the drug puromycin, whose incorporation into nascent proteins is a highly reliable readout of protein synthesis levels. The addition of specific chemical inhibitors allows the estimation of glucose dependence, mitochondrial dependence, glycolytic capacity, and fatty acid and amino acid oxidation capacity. Flow cytometry analysis, using a fluorescent antibody against puromycin, defines simultaneously the ex vivo phenotype and the energy metabolism of multiple cell populations in parallel [74]. Using this methodology, we found that γδ T cell subsets exhibit clearly distinct metabolic profiles that are imprinted during γδ thymic development and maintained in peripheral lymphoid organs and within tumors [16]. On one hand, γδ^IFN^ T cells are highly glycolytic, consistent with reports showing that glycolysis is required for the production of IFN‐γ by NK cells [75] and CD8^+^ T cells [76]. On the other hand, γδ^17^ T cells have higher mitochondrial mass (and membrane potential) and strongly rely on OXPHOS [16], similarly to CD4^+^ Th17 cells [77]. We found this dichotomy in γδ T cell subsets to have a transcriptional basis, including the segregation of two master regulators: *Nrf1*, which orchestrates mitochondrial DNA transcription, was enriched in γδ^17^ T cells; and *Myc*, which controls glycolysis, was highly overexpressed in γδ^IFN^ T cells [16].

### Metabolic modulation of γδ T cell activities in the tumor microenvironment

It is well known that tumors adopt characteristics, including selected metabolic advantages linked to intense proliferation, that promote T cell hyporesponsiveness or dysfunction to escape immunity [78]. Tumor‐infiltrating immune cells can be shaped by nutrient availability in the TME. For example, glycolysis has been shown to regulate IFN‐γ production in T cells [79]; and antibody‐mediated blockade of the PD‐1/PD‐L1 immune checkpoint restored glycolytic and effector functions of tumor‐infiltrating T cells [80]. While performing aerobic glycolysis (“Warburg effect”), tumor masses heavily consume glucose, which deprives T cells and alters their metabolism and functionalities; thus, metabolic competition in the TME is a driver of cancer progression [80]. Consistently, resistance to adoptive T cell therapy is observed in patients with an increased tumor glycolysis [81]. Furthermore, it has been shown that high levels of lactate in the TME lead to diminished antitumor immunity by increasing the apoptosis of naïve T cells and inhibiting effector T cell functions [82, 83]. Importantly, amino acid restriction such as serine, glutamine or glycine deprivation also leads to an impaired function of CD8^+^ T cells and NK cells [84, 85, 86].

**Figure 3:**
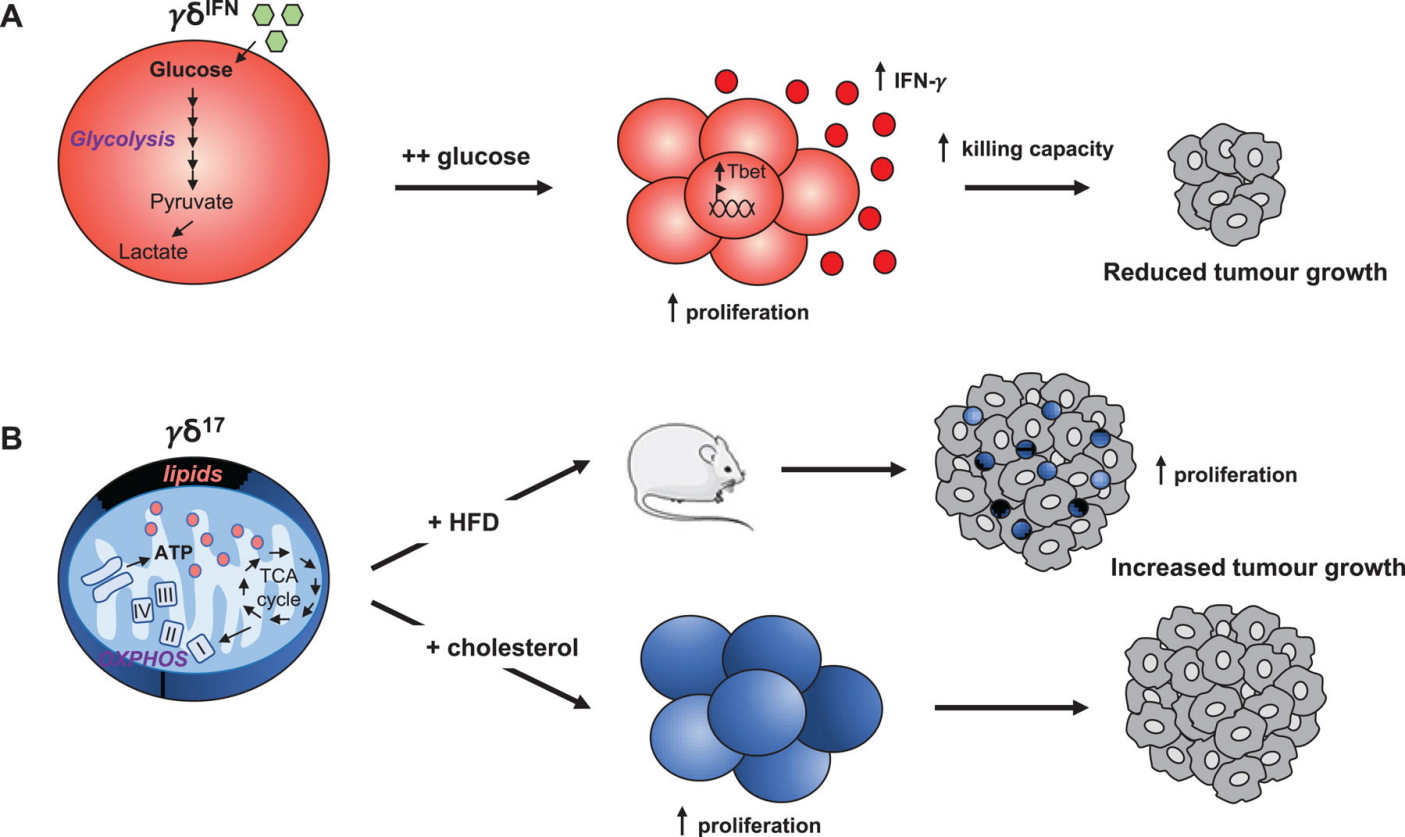
Distinct metabolic cues control γδ T cell subset expansion and functions in the TME. (A) γδ^IFN^ T cells uptake more glucose and employ aerobic glycolysis. γδ^IFN^ T cells supplemented with high dose of glucose exhibit increased (i) proliferation, (ii) Tbet expression, (iii) IFN‐γ production and (iv) killing capacity against cancer cells. Consequently, the adoptive transfer of glucose‐”boosted” γδ^IFN^ T cells substantially reduces tumor growth. (B) γδ^17^ T cells display high lipid uptake and intracellular lipid storage; and engage mitochondrial oxidative metabolism. γδ^17^ T cells are expanded in lipid‐rich environments such as in tumor‐bearing obese mice fed with high‐fat diet (HFD) leading to increase tumor growth. Likewise, cholesterol supplementation promotes γδ^17^ T cell proliferation and increases tumor growth after adoptive transfer.

We have addressed the impact of the metabolic differences between effector γδ T cell subsets on their activities in the TME, particularly upon adoptive cell transfer. Given the high(er) glycolytic activity of γδ^IFN^ T cells, we explored the effect of glucose supplementation on their antitumor functions in vitro and in vivo. We found high (10‐fold higher concentration of) glucose to enhance γδ^IFN^ cell proliferation, IFN‐γ production, and antitumor cytotoxicity in vitro [16]. We did not consider injecting glucose directly into tumors given that it would likely benefit cancer proliferation progression [80]. Instead, we provided it to γδ^IFN^ T cells during a short (5 h) incubation before adoptive transfer (intra‐tumoural injection); this resulted in a substantial reduction in breast tumor growth in vivo (Fig. 3).

By contrast, high glucose was detrimental to γδ^17^ T cell proliferation, as well as to their generation in fetal thymic organ cultures [16]. We therefore questioned which other metabolic resources could fuel γδ^17^ T cells and their activities in the TME. We found that lipids, including palmitate and cholesterol, are preferentially uptaken by γδ^17^ T cells, which become significantly expanded in tumors from high‐fat diet (HFD)‐treated obese mice (Fig. 3). Consistent with this, short‐term (5h) cholesterol treatment in vitro leads to increased proliferation of γδ^17^ T cells and, upon adoptive transfer, cholesterol‐treated γδ^17^ T cells substantially increased (compared to controls) breast tumor growth in vivo (Fig. 3). These data, suggesting that a lipid‐rich environment boosts γδ^17^ T cells to support tumor growth, provide a new mechanism to the body of evidence linking obesity and cancer [87], including via immunometabolism [88, 89].

In sum, we propose that the relative abundance of glucose versus lipids is a key determinant of the balance between protective γδ^IFN^ T cells and pathogenic γδ^17^ T cells in the TME.

### Conclusions and implications for human disease

Although there are additional pro‐ versus antitumor mechanisms deployed by γδ T cells (Fig. 2; reviewed in [10]), here we focused on those contributed by γδ^IFN^ and γδ^17^ T cells. The concept of a functional dichotomy among γδ T cell subsets in tumor immunity may have important implications for development of γδ T cell‐based cancer immunotherapies. On that road, an outstanding question to resolve using preclinical models is how the balance between γδ^IFN^ and γδ^17^ T cells is regulated, particularly in the TME, toward promoting antitumor immunity. Our most recent research suggests that metabolism may provide some answers, namely by limiting lipids in the TME; and favoring aerobic glycolysis in γδ T cell‐based adoptive cell therapy (ACT) products/ strategies.

We believe that increased understanding of tumor and immune cell metabolism may be a powerful tool for the development of next‐generation immunotherapies. Targeting the metabolism of immune cells through the modulation of key metabolic pathways and/or dietary interventions in order to enhance their antitumor functions may improve cancer treatment [90]. An interesting avenue to explore within immunometabolism is the impact of different amino acids, since the TME also creates competition between tumor and T cells for essential amino acids. For example, arginine is depleted by several tumors, which leads to inhibition of T cell activation and an immunosuppressive environment [91, 92]. Thus, optimizing arginine concentrations has a direct impact on the metabolism and survival of T cells [92]; and one may consider providing additional arginine to cancer patients with reduced plasma arginine levels. Another example is glutamine, as glutaminase activity, which converts glutamine to glutamate, is required to promote Th1 and cytotoxic CD8^+^ T cell functions [93]. Finally, it has also been shown tumor cells express IDO, an enzyme that depletes tryptophan and thus inhibits T cell proliferation [94]. Interestingly, it has been recently shown that IDO inhibitors enhanced human γδ T cell cytotoxicity against pancreatic ductal adenocarcinoma [95]. Clinical trials with IDO inhibitors in advanced solid tumors are ongoing.

We find the context of ACT particularly interesting to modulate T‐cell metabolism without having undesired effects on tumor cells. For example, modulating activated T cells to have memory‐like metabolism, weighted toward oxidative phosphorylation (OXPHOS) and fatty acid oxidation [90], or supplementing with high doses of glucose to increase the cytotoxic activity of Th1, CD8^+^ or γδ T cells, may greatly improve antitumor responses in vivo (upon ACT). Thus, enhancing “metabolic fitness” of effector T cells with metabolic resources or pharmacologic agents targeting key metabolic pathways may improve the clinical efficacy of T cell‐based therapies. Like glucose supplementation, the addition of specific amino acids to expansion protocols for ACT or even as dietary supplement may also regulate γδ T cell (subset) functions in the TME. This hypothesis deserves furthern investigation.

It is of interest to combine metabolic modulation with immune checkpoint blockade to improve cancer treatment. In fact, both CTLA‐4 and PD‐1 inhibit T‐cell activation via suppression of metabolic activity, including the downregulation of AKT phosphorylation, and decreased glucose and amino acid uptake [96, 97]. On the other hand, CTLA‐4 and PD‐1 also interfere with CD28 signaling [98], which is known to sustain glycolysis and to prime mitochondria during T cell activation [99]. Interestingly, it has been recently shown that in vivo triggering of 4‐1BB costimulation, in combination with PD‐1 blockade, results in robust antitumor immunity in a B16 melanoma model [100]. Thus, metabolic modulation and immune checkpoint blockade may constitute a promising combination to increase the efficacy of cancer immunotherapy.

Although not detailed in this review, another major factor to consider is the microbiome, based on a very interesting link recently uncovered between commensal microbiota and pro‐tumoural γδ^17^ T cells in mouse models of lung cancer. The bacteria were shown to induce the production of IL‐1β and IL‐23 by myeloid cells, which triggered high proliferation and activation of Vγ6^+^Vδ1^+^ tissue‐resident γδ^17^ T cells that supported tumor growth [22].

Pre‐clinical models are also extremely valuable to dissect the cellular crosstalks that may control the balance between tumor‐infiltrating γδ^IFN^ and γδ^17^ T cells. In this regard, we have demonstrated that neutrophils can suppress γδ^17^ T cell responses, while not affecting γδ^IFN^ T cells, through ROS‐mediated oxidative stress [34]. Other positive or negative regulators of γδ T cell subsets in the TME, including regulatory T cells [101], should be better defined in future research.

Ultimately, one should consider the relevance of such findings on mouse γδ T cells to human cancer, particularly as the correspondence between murine and human γδ T cell subsets is not straightforward. Namely, the Vγ‐based definition of murine γδ T cell subsets does not apply to humans, where the main sub‐populations are defined by the Vδ (segment) usage of their TCR [102]. Thus, in humans, Vδ1^+^ T cells are enriched within healthy (as well as malignant) tissues, whereas Vδ2^+^ T cells (mostly Vγ9^+^) predominate in the peripheral blood [14]. Both subsets have been clearly implicated in cancer immunity [10, 11, 103], especially given their very strong cytotoxic type 1 (γδ^IFN^) biases [10, 104]. In fact, γδ^17^ T cells are much less frequent in humans than in mice, and the presence of human γδ^17^ T cells in cancer is somewhat controversial. While Wu and colleagues reported that human γδ T cells are the major cellular source of IL‐17 in colorectal cancer [28], a more recent study demonstrated by using three different flow cytometry gating strategies that the majority of IL‐17‐producing leukocytes in colorectal cancer were CD3^+^ but not Vδ1^+^ or Vδ2^+^ T cells, suggesting they are mostly Th17 cells [29]. Nonetheless, γδ^17^ T cell infiltration was shown to correlate with poor survival in gallbladder cancer in humans, in sharp contrast with γδ^IFN^ T cells [105]. Thus, the balance between γδ^IFN^ and γδ^17^ T cells may be informative also in human cancer, although its value as a potential biomarker remains to be established.

## Author contributions

N.L. and B.S.‐S. conceived and wrote the manuscript.

## Conflicts of interest

B.S.‐S. is co‐founder and shareholder of Lymphact S.A., a start‐up company acquired by GammaDelta Therapeutics (London, UK). N.L. declared no commercial or financial conflicts of interest.

**Abbreviations**
γδ: gamma delta
ACT: adoptive cell therapy
DETC: dendritic epidermal γδ T cell
HFD: high‐fat diet
IFN‐γ: interferon‐gamma
IL‐17: interleukin‐17
LN: lymph node
MCA: methylcholanthrene
MDSC: myeloid‐derived suppressor cell
OXPHOS: oxidative phosphorylation
SCENITH: single cell metabolism by profiling translation inhibition
SPM: small peritoneal macrophage
TCR: T‐cell receptor
TME: tumor microenvironment
VEGF: vascular endothelial growth factor
